# Topical Probiotic Hydrogels for Burn Wound Healing

**DOI:** 10.3390/gels10090545

**Published:** 2024-08-23

**Authors:** Tavinda Arshad, Varsha Mundrathi, Victoria E. Perez, Jeilyn M. Nunez, Hyunah Cho

**Affiliations:** Industrial Pharmacy, Department of Pharmaceutical Sciences, College of Pharmacy and Health Sciences, St. John’s University, Queens, NY 11439, USA; tavinda.arshad23@my.stjohns.edu (T.A.); varsha.mundrathi23@my.stjohns.edu (V.M.); victoria.perez19@my.stjohns.edu (V.E.P.); jeilyn.nunez19@my.stjohns.edu (J.M.N.)

**Keywords:** probiotics, topical, hydrogels, burn wound, wound healing

## Abstract

Hydrogels have increasingly been used to enhance the effective healing of various wounds, including burn wounds. Similarly, the application of probiotics has recently been explored in wound healing and skin repairs. While probiotics have been consumed to provide therapeutic effects that aid with improving gut health, topical applications have been found to accelerate wound healing both in vitro and in vivo. For wounds that have complex healing mechanisms, such as burn wounds which depend on factors such as the depth of the burn, size of the afflicted area, and cause of the injury, probiotics with or without conventional therapeutic agents topically delivered via hydrogel technology are proven to be effective in the recovery of the damaged skin. This article aims to investigate the microorganisms present in the human skin microbiome and observe the effects of probiotics delivered by hydrogels on burn wound healing.

## 1. Introduction

The human skin biome is known to play a key role in maintaining skin homeostasis (e.g., preventing fluid loss), defending the body against exogenous pathogens (e.g., antimicrobial effect), and modulating the immune system [1]. Probiotics have gained popularity for improving skin health and modulating the human skin biome in diseased or injured skin. For example, coagulase-negative *Staphylococcus* (CoNS) populated in healthy human skin is reported to produce antimicrobial activity and provide host defense against pathogens such as *Staphylococcus aureus* [2]. *Staphylococcus aureus* is known to be associated with atopic dermatitis and CoNS is deficient in the skin of atopic dermatitis patients. Therefore, the topical reintroduction of CoNS suppresses *Staphylococcus aureus* in the skin and potentially provides therapeutic effects in atopic dermatitis. In the last few years, topical probiotics have been researched on burn wound healing and have been shown to accelerate the wound healing process and provide antimicrobial and anti-inflammatory effects. Yet, topical probiotic research is in its infancy and topical probiotic delivery methods have not been well established. In this article, the microorganisms present in the human skin microbiome are summarized and the recent articles that report the therapeutic effects of topical probiotic hydrogels on burn wound healing are reviewed.

### 1.1. Human Skin Microbiota

The human skin is home to a complex ecosystem comprising different microorganisms such as bacteria, fungi, and viruses, forming what is known as the skin microbiota [3]. The skin microbiota is fundamental to skin physiology and immunity, serving as a physical barrier to protect the skin against pathogens and modulating the immune system [4]. The microbiome itself is a layer just above the epidermis, which is the outermost surface of the skin, and can vary from region to region on the body, although the microbes occupy several layers in the skin. It is known that changes in environment, such as moisture and pH, can affect the microorganisms living on a specific part of the skin [5]. For instance, the microbiota on the face thrive in a more oily environment, while those on the forearms prefer a drier microenvironment [6]. These variable environments are crucial to ensuring a healthy, functional microbiome, as imbalance of the inhabitants occupying the space can be detrimental to health. Estimating a baseline and the composition of the microbiota at different sites of the human skin is essential to elucidate how a change in the normal microbiome, known as dysbiosis, causes skin disorders. The human skin microbiota is the organ possessing the most microorganisms on the body, after the gut. It consists of hundreds of bacterial species, with both commensal (harmless) as well as pathogenic (harmful) bacteria living together harmoniously, until imbalance amongst the different strains leads to dysbiosis [7]. The microbes on the skin also signal immune responses upon receiving a physical injury and result in a change in the microbial flora in order to reduce inflammation, although the presence of the wound often allows pathogenic bacteria to invade the vicinity. This is driven mostly by probiotic bacteria on the skin, which will be discussed further in this paper. In fact, various probiotic microorganisms play a key role in managing various skin disorders (Table 1). For instance, *Vitreoscilla filiformis*, *Streptococcus thermophilus*, *Lactobacillus johnsonii*, and *Bifidobacterium* species are known to manage atopic dermatitis (eczema) [5]. *Bifidobacteria infantis* and *Lactobacillus pentosus* are involved in managing psoriasis [5]. *Bifidobacterium breve BR03* and *Lactobacillus salivarius* are reported to progress rosacea [5]. Conversely, damage to the skin can allow an influx of harmful pathogens into the body, and these tend to cause infection of the wound, thereby disrupting the healing process and often leading to various disease states. The addition of probiotics during treatment of a wound can aid in a faster recovery, as they can help prevent infection and reduce inflammation, thereby expediting the wound healing process [7].

### 1.2. Prebiotic, Probiotic, Postbiotic

Over the past few years, it has been found that several diseases and symptoms occur due to disturbances in the gut microbiota [9]. While various medicines can target specific sites of action, they often cause side effects that result in imbalances within the gut, thereby exacerbating unfavorable circumstances which may lead to dysbiosis in the skin, alongside other organs. Skin problems are the easiest to identify since they are external, but it is important to consider the internal organs which may similarly be afflicted by changes in the gut. Thus, it is essential to understand the root cause of the problem with a thorough understanding of the skin microbiome alongside gut microbiota and how the two work together to promote the overall health of a body.

Probiotics, prebiotics, and postbiotics have thus been investigated in recent times to be optimized alongside medicine used for general treatment of diseases. As stated by the Food and Agriculture Organization (FAO) and World Health Organization (WHO), “probiotics are live microorganisms which, when administered in adequate amounts, confer health benefits on the host” [10]. Probiotics are generally consumed as part of the diet, and can be found in fermented foods such as yogurt, kefir, and cheese [11] or unfermented foods such as cereal and chocolate [12]. Upon consumption, they are known to improve health in a number of ways, such as by aiding digestion, interacting with immune cells, and moderating the bacteria present in the gut. They can restructure the microorganisms present, supporting immunity and thereby preventing pathogenic attack in the intestine [13]. Certain probiotic strains of *Lactobacillus* and *Bifidobacterium* can also increase production of ceramides, which are crucial elements of the skin’s protective barrier that retain water, preventing external pathogens from entering the body. A combination of internal and external probiotics works to boost the body’s immunity, hence preventing the onset of disorders such as cardiovascular diseases or even diabetes. One notable probiotic strain could be those of lactic acid bacteria, such as *Lactococcus* and *Lactobacillus*, which are present in foods such as sourdough bread or kimchi.

Unlike probiotics, which are living organisms, prebiotics are simply ingredients that are not digestible by the body, but which nonetheless enhance the growth of probiotics and microorganisms in the gut [13]. According to the International Scientific Association for Probiotics and Prebiotics, “prebiotics are substances that can be selectively used and transformed by the host intestinal flora under the premise that they are beneficial to host health”. They are a food source for these organisms and are generally indigestible polysaccharides found in sources such as fruit peels and juices, seeds and plants used in traditional medicines. The presence of prebiotics is known to increase metabolism and boost immunity by preventing pathogenic growth in the gut. They are also able to regulate the pH of the skin microbiota, allowing for the support of the external probiotics present and, consequently, strengthening the skin barrier. There are many substances which can be considered to have a prebiotic effect, including lipids [14]. These provide a healthy environment for beneficial microbes on the skin to proliferate, and fermentation of prebiotics can produce short-chain fatty acids, which can alter pH, stimulate the immune system, and increase circulation of blood to other organs [15].

Lastly, postbiotics are by-products of probiotics which contribute to gut health [9]. They are derived upon the death of probiotics, and thus can be considered inanimate substances, and can be composed of proteins, cell fragments, or microbes [12]. While probiotics are living organisms, postbiotics can be considered to be the deceased version of a probiotic. However, death alone does not make a postbiotic. The inanimate substance must often be activated by pH, heat, and other environmental factors to provide beneficial effects to the gut. Thus, probiotics must be carefully prepared into postbiotics for an efficacious effect to be observed. The discovery of postbiotics is relatively new, but they are known to assist with enhancing health in tandem with probiotics and prebiotics. According to studies by Callewaert et al. in 2021, skin microbiome modulation strategies can be implemented to maintain or even eliminate pathological microbes which can improve several dysbiosis (skin) conditions [16]. The term bacteriotherapy has been revived to explain the use of probiotics and postbiotics in skin therapy.

Postbiotics, prebiotics, and probiotics have been known to successfully moderate the microbiome orally, with *Lactobacillus*, *Bifidobacterium*, *Enterococcus*, *Lactococcus*, *Streptococcus*, *Bacillus*, and yeast species such as *Saccharomyces boulardii* being the most frequently used microbes [6]. The oral intake of these microorganisms was found to improve the barrier of the gut, reduce the presence of pathogens, support immunomodulation, and assist the immune response of other organs. An example of a food that consists of probiotics, prebiotics, and postbiotics is baby formula. Infants consuming this fermented formula were found to moderate the gut microbiota, thereby reducing inflammation, diarrhea, and pH changes in the stool. Postbiotics may also be considered as a food supplement over probiotics, since they will have longer shelf-life due to enhanced stability. Compared to oral probiotics, topical probiotic research on skin repair and wound healing is still in its infancy, although many protocols and guidelines can be adapted from the existing oral studies [6].

## 2. Topical Probiotics for Burn Wound Healing

Skin, the integumentary system, is the body’s most extensive and primary protective organ, and serves as the physical barrier against the external environment and disease-causing pathogens. As the body’s first line of defense, skin can be damaged upon contact with heat and cause wounds. High temperatures, electricity, radiation, and chemicals can result in burn wounds, necrosis, and deep tissue damage. The severity of the burn depends on several factors, such as the temperature at which the injury occurred and the depth of the wound. Chemicals such as strong acids cause damage to the skin. Similarly, when electric current or voltage is passed through the skin, the current disrupts the skin barrier, causing skin injury [17]. Immediate colonization by the patient’s normal skin flora (i.e., *Staphylococcus aureus* and *Streptococcus pyogenes*) often occurs following injury. Subsequent colonization by the patient’s own gut flora adds to the complex microbial ecology on the burn wound surface shortly thereafter [18]. As a result, burn wounds become colonized with microorganisms [19]. The nature and degree of the thermal injury along with the type and amount of the microorganisms appear to influence the progression of the invasive wound infections. The invasion of microorganisms into the wounds causes infection. Burn wounds have open layers of the skin that are susceptible to infections, providing access to microbial invasion, allowing them to colonize and cause severe infections, potentially leading to sepsis. Several factors, such as age, medical conditions, hygiene, depth of burn, and delayed treatment, lead to severe infections due to microbial exposure to the wounds [20]. Both Gram-positive bacteria, such as *Staphylococcus aureus* and *Staphylococcus pyogenes*, and Gram-negative bacteria, such as *Klebsiella species*, *Escherichia coli*, and *Pseudomonas aeruginosa*, cause infections at the site of burn wounds. Colonization of these bacteria at the burns leads to severe infections and might lead to death if not treated properly. *Staphylococcus aureus* invasion at the site of the burn causes systematic infections and sepsis. In contrast, *Pseudomonas aeruginosa* is the most pervasive bacterium found at wound sites, potentially leading to sepsis and death over time. Macedo et al. demonstrated that *Staphylococcus aureus* (20.5%) was the prime organism causing infection in burn wounds, followed by *Pseudomonas aeruginosa* (11.4%) [21]. Fungal infections arise due to the prolonged use of broad-spectrum antibiotics. Fungi such as *Candida albicans* are pathogenic and cause significant mortality due to profound infections in burn patients [22].

**Figure 1:**
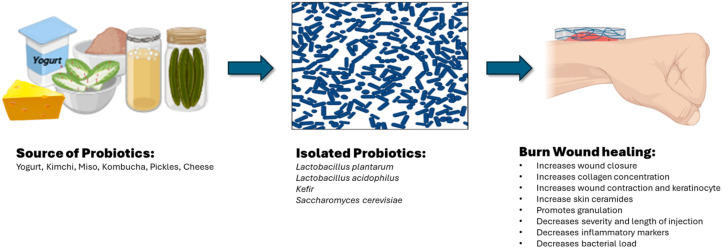
Properties of isolated probiotics for burn wound healing (created in BioRender, https://www.biorender.com/, 8 August 2024).

Local burn wound care aims to protect the wounded skin surface, provide a moist environment, limit wound progression, and promote wound healing and skin repair [23]. Conventional local burn wound care includes dressings (e.g., Acticoat^®^, smith & nephew, Memphis, TN, USA), antimicrobial ointments/gels (e.g., polysporin, mupirocin, silver sulfadiazine, chlorhexidine), plant/food-based ointments/gels (e.g., aloe vera, honey), and biologic grafts (e.g., allogenic skin grafts, human amnion). However, the emerging treatments are resistant to conventional approaches; specifically, the systemic and topical antibiotic variety have challenged the treatment of burn wounds and infections. Topical probiotics have arisen as an alternative in burn wound treatments. It has been noted that the absence and/or imbalance of skin microbiota plays a key role in both the rate and process of wound healing [24]. The introduction of exogenous bacteria followed by the imbalance between endogenous (host) skin microbiota and exogenous bacteria can hinder the wound healing process and cause skin infection to occur. Probiotics promote numerous health benefits by improving gut health and enhancing immune functions. Several studies proved that topical probiotics show promising results in wound healing, including burn wounds. Probiotics are live microorganisms that show antimicrobial/antibacterial properties and contribute to the regulation of inflammation and infection, allowing for improvement in the wound healing process [25]. The topical application of probiotics has been suggested to be an alternative to antibiotics to treat infections. According to the studies performed on animal models, probiotics have shown prominent results in treating surgical lesions and burn wounds [7]. For instance, probiotics have been shown to act as an antibacterial agent against Methicillin-resistant *Staphylococcus aureus* (MRSA) and as an antimicrobial agent, accelerating a burn wound healing process [24]. *Staphylococcus aureus* is known to adhere with the epidermal keratinocyte cells via the α5β1 integrin to proliferate. Probiotics reduce keratinocyte cell death by preventing the pathogen from adhering to the integrin’s binding sites on the skin cells. As mentioned above, two other common pathogens isolated from burn wounds are *Pseudomonas aeruginosa* and *Streptococcus pyrogenes* [7]. These common bacteria are known to produce biofilms, a complex community embedded in a polysaccharide matrix. Such bacteria growing in biofilms are resistant to various antibiotics and antiseptics. Probiotics play a preventative role against biofilm development and augment cell proliferation and angiogenesis. Here, we listed a few probiotics (i.e., *Lactobacillus plantarum*, *Lactobacillus acidophilus*, *kefir*, and *Saccharomyces cerevisiae*) reported to accelerate burn wound healing and skin rejuvenation (Figure 1).

### 2.1. Lactobacillus plantarum

In a pre-clinical study, Satish et al. found that a single inoculation of *Lactobacillus plantarum* (3 × 10^8^ CFU, ATCC 10241 strain grown in MRS broth) inhibited *Pseudomonas aeruginosa* by c.a. 37% on thermal (100 °C) skin burn wounds created on the dorsum of male Dutch Belted rabbits [26]. This probiotic therapy inhibited the injury-induced accumulation of type I collagen mRNA by c.a. 50%, resulting in a significantly lessened amount of total collagen protein than those in non-treated wounded skin and unwounded skin. Interestingly, *Lactobacillus plantarum* appeared to alter the type of collagen alignment after burn injury from mature type I collagen into immature type III collagen, noting that type III collagen is favored in wound healing and skin repair. It is important to note that *Lactobacillus plantarum* itself did not elicit notable inflammatory or fibrotic effects in the host skin tissues.

Peral et al. carried out a clinical study to determine whether the topical application of *Lactobacillus plantarum* (10^5^ CFU, ATCC 10241 strain/mL grown in MRS broth) can provide an alternative to treat infected second-degree burns and non-infected and infected third-degree burns compared to silver sulphadiazine treatment [27]. Eighty male and female patients from the plastic surgery and burn unit were included in the study. A whole culture was spread on a gauze pad and then applied to the burn (1 mL/cm^2^). Wound bacterial pathogens were identified as *Staphylococcus aureus*, *Pseudomonas aeruginosa*, *Staphylococcus epidermidis*, *Enterobacter cloacae*, *Klebsiella pneumonia*, and *Enterococcus faecalis*. Clinical follow-ups were made for 10 days to determine the degree of active granulation tissue formation (shown as bright red tissue in the bed of the burn wound). Changes in graft skin color, existence of blisters, skin adhesions, death of the graft, hemorrhage, blood clots, and infections were monitored. There was no skin graft required for healing when treated with *Lactobacillus plantarum*. Decreased bacterial loads (less than 10^5^ bacteria per 1 g of tissue) were measured when treated with either topical *Lactobacillus plantarum* or silver sulfadiazine in patients with delayed second-degree burns. Complete healing was achieved in 75% of patients treated with *Lactobacillus plantarum* and 77% treated with silver sulphadiazine. There was a decrease in bacterial load and promoted granulation tissue in the patients with delayed third-degree burns when treated with *Lactobacillus plantarum*. Complete healing was achieved in 75% of patients treated with *Lactobacillus plantarum* and 65% treated with silver sulfadiazine in these patients. The percentage of skin graft required between both treatments was 90% in patients with delayed third-degree burns. Based on clinical signs, symptoms, and standard blood assays, invasive infection caused by pathogens from the burn wound (sepsis) was not observed in any of the patients before, during, or after either treatment. Clinical observation also displayed a similar quality of healing and re-epithelialization between both treatments.

In a Phase I clinical trial, Soleymanzadeh et al. prepared ointment containing *Lactobacillus plantarum* bacteria-free supernatant and applied it to 18 patients with deep second-degree burns on two feet or two hands [28]. One hand or one foot (left) was treated with wound washing (normal saline) followed by silver sulfadiazine ointment and dressing for 7–10 days, whereas the other hand or foot (right) was treated with wound washing (normal saline) followed by ointment containing *Lactobacillus plantarum* and dressing for 7–10 days. In the *Lactobacillus plantarum*-treated group, *Pseudomonas aeruginosa* was isolated from 4 out of 18 (6 out of 18 from the silver sulfadiazine-treated group). A total of 12 out of 18 were free from bacteria (9 out of 18 from the silver sulfadiazine-treated group). Regarding the evaluation of resistance to antibiotics, the resistance to gentamicin was lower in *Pseudomonas aeruginosa* strains isolated from the *Lactobacillus plantarum*-treated group compared to those isolated from the silver sulfadiazine-treated group (9 vs. 12). Patients from both groups were required to proceed with skin grafting; however, the rate of graft rejection in the *Lactobacillus plantarum*-treated group was 0 out of 18, whereas that in the silver sulfadiazine-treated group was 4 out of 18. The authors stressed that *Lactobacillus* strains are a great treatment option to treat burn wounds as these strains are safe, do not produce virulent factors, and induce significant anti-inflammatory effects.

### 2.2. Lactobacillus acidophilus

Barzegari et al. prepared ointment carrying *Lactobacillus acidophilus* (0.8 × 10^10^ to 0.8 × 10^11^ CFU, ATCC 4356 strain cultured in MRS broth) to evaluate the effect of topically applied *Lactobacillus acidophilus* on the second-degree burn (97 °C) wound healing process in male Wistar rats [29]. Four applications of ointment carrying *Lactobacillus acidophilus* (days 1, 3, 7, and 14) led to a better macroscopic result, demonstrating 123% of wound healing for treatment group (107% healing for vehicle group). More striking results were shown in microscopic evaluations. On the 14th day of application, based on histopathological analyses, *Lactobacillus acidophilus* induced much less inflammatory response, accelerated tissue granulation, and notable re-epithelialization.

Jebur et al. isolated pathogens from 50 patients who were admitted to a local hospital due to burn wounds, and isolated pathogens were treated with *Lactobacillus acidophilus* (10^8^ cells/mL) in vitro [30]. From the patient’s samples, the authors were able to identify eight bacterial isolates, which were *Pseudomonas aeruginosa*, *Escherichia coli*, *Enterobacter* spp., *Klebsiella* spp., *Proteus* spp., *Staphylococcus aureus*, *β-hemolytic Streptococci*, and *Staph*, *epidermidis*. These bacterial isolates were universally susceptible to *Lactobacillus acidophilus*, except *Klebsiella* spp., showing a 75% susceptibility. For instance, *Escherichia coli* was highly sensitive to *Lactobacillus acidophilus* with 100% susceptibility, whereas amikacin (95%), ciprofloxacin (90%), and azithromycin (95%) showed less than 100% susceptibility. The authors stated that *Lactobacillus acidophilus* exhibited an antibacterial effect by interacting with toll-like receptor-2 (TLR-2) which recognizes bacterial lipoproteins, lipoteichoic acid, and zymosan.

Khan et al. isolated *Lactobacillus acidophilus* (10^11^ CFU/mL) from fermented yogurt and applied it topically on the wounds of 45 male Wister albino rats [31]. There was a significant increase in the thickness of the epidermis and dermis layers, indicating a significant skin healing effect, which was superior to topical application of neomycin.

### 2.3. Kefir

Kefir is a natural probiotic containing a mixture of bacteria and yeasts and known to be antioxidant, anti-inflammatory, and antimicrobial. Oryan et al. reported that the most dominant strains in kefir were *Lactobacillus kefiranofaciens*, *Lactobacillus kefiri*, *Lactobacillus plantarum*, *Lactobacillus parakefiri*, *Lactococcus lactis*, *Saccharomyces cerevisiae*, and *Saccharomyces unisporus* [32]. Kefir was effective as an antibacterial agent in vitro against *Pseudomonas aeruginosa*. The effect of wound closure induced by Kefir was shown to be superior to that of silver sulfadiazine, through inducing fibroblast cell migration. Kefir enhanced re-epithelialization and decreased scar formation in vivo on male Sprague-Dawley rats with burn wound (100 °C). Kefir also improved the remodeling stage by reducing interleukin-1 beta (IL-1β) and basic FGF content and increasing transforming growth factor beta 1 (TGF- β1) and hydroxyproline contents.

Rodrigues et al. tested 70% Kefir gel on antimicrobial and healing activities against several bacterial species in vitro [33]. Kefir grain used for this study contained a significant number of *Leuconostoc* spp., *Lactobacillus lactis*, *Acetobacter* spp., *Saccharomyces cerevisae*, *Kluyveromyces marxianus*, and *Kluyveromyces lactis*. *Staphylococcus pyogenes* was the most sensitive to kefir followed by *Staphylococcus aureus* and *Staphylococcus salivarius*, *Staphylococcus typhimurium*, *Candida albicans*, and *Listeria monocytogenes. Pseudomonas aeruginosa* and *Escherichia coli* were the least sensitive to kefir. Kefir showed the most significant antimicrobial effects on *Staphylococcus aureus*, *Pseudomonas aeruginosa*, and *Candida albicans* compared with some antibiotics such as ketoconazole, ampicillin, azithromycin, ceftriaxone, and oxacillin. The skin wound treated with 70% kefir gel showed a well-developed granulation of the epithelium and the significant areas of neovascularization in vivo.

Yildiz et al. explored the wound recovery properties of kefir on burn wounds that were infected with *Staphylococcus aureus*, *Pseudomonas aeruginosa*, and *Escherichia coli* [34]. The infected burn wound was treated with sterile pads dressed in kefir. Kefir dressing appeared to accelerate epithelial proliferation, vascular proliferation, fibroblastic proliferation, and collagenization in the infected second-degree wounds on Swiss albino/Balb-c mice. The authors found that infected burn wounds treated with kefir wound dressing developed a thick and well-developed epidermal layer with significantly lower inflammatory cell infiltrates.

### 2.4. Saccharomyces cerevisiae

Oryan et al. evaluated the concurrent use of *Saccharomyces cerevisiae* (MYA796 strain grown in YM broth) and collagen hydrogel/scaffold in burn wound (100 °C) healing [35]. The wound was first injected subcutaneously with a suspension of *Saccharomyces cerevisiae* (3 × 10^6^ CFU/300 μL) and then covered by collagen hydrogel/scaffold dressing. The epidermal layer was completely formed, and rejuvenation of skin appendages and hair follicles was monitored. The inflammatory response and tissue granulation was found to be low and re-epithelialization occurred at day 22 post-treatment. The treated wound showed a significantly lower count of inflammatory cells. It also showed the best cosmetic appearance, presenting the smallest wound size and the skin condition (e.g., linear stiffness, ultimate tensile load and strain) closest to the unwounded normal skin on day 22.

## 3. Probiotic Hydrogels for Burn Wound Healing

Traditional bandages, pads or gauzes for burn wounds are not sufficient to provide the moist environment for effective wound healing [36]. With the development of biomaterials, advanced forms of wound dressings have emerged as alternatives, such as nanofibers, films, and hydrogels. Hydrogels are a three-dimensional network of natural or synthetic polymers formed via physical or chemical crosslinking. Hydrogels are bioadhesive and known to mimic extracellular membranes in structure, rheology, and chemistry. Such intrinsic potential makes hydrogels an attractive platform for wound healing (Table 2). Hydrogels are generally hydrophilic and high in water content. The high level of the water content provides cooling and soothing effects to the wounded area, reducing pain. The semi-solid property of hydrogels possesses mechanical strength similar to skin and the diffusion properties of a liquid [37]. Unlike other dosage forms such as ointments, creams, and gauzes, hydrogels can provide coverage of the wound surface, even for wounds with complex/irregular shape, and maintain the moist healing environment which is widely accepted as the key factor of promoting rapid burn wound healing (Table 2).

**Figure 2:**
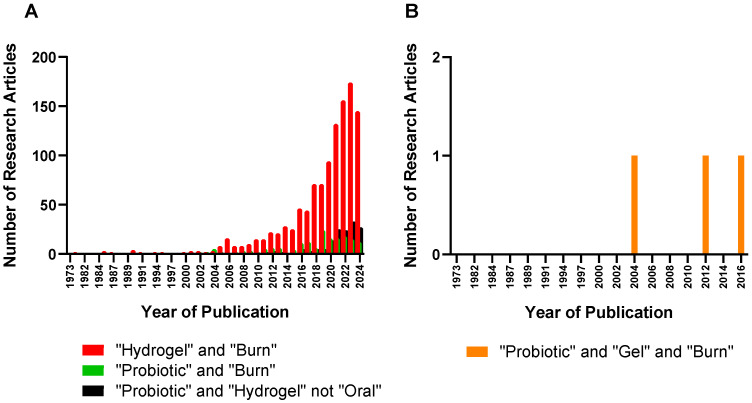
The number of research articles concerning the development of hydrogels and the delivery of probiotics for burn wound treatments. (**A**) Number of research articles with the keywords “hydrogel” and “burn”, “probiotic” and “burn”, and “probiotic” and “hydrogel” not “oral” (**B**) Number of research articles with the keyword “probiotic” and “gel” and “burn” (source: PubMed. Date: 8 August 2024).

**Figure 3:**
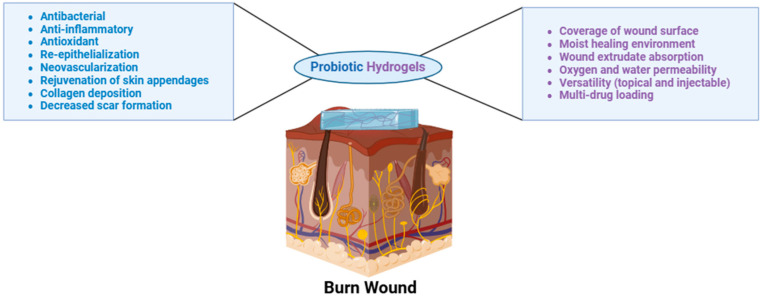
Properties of probiotic hydrogels for burn wound healing (created in BioRender, https://www.biorender.com/, 20 August 2024).

Hydrogels can absorb wound exudates and maintain good oxygen and water permeability for better wound healing, thus promoting fibroblast proliferation and keratinocyte migration [38,39], Hydrogels are capable of delivering multiple therapeutic agents to the wounded skin for extended periods via topical application or injections around the affected areas. Hydrogels used for wound healing and/or burn wound healing are most often composed of natural polymers such as alginate, chitosan, hyaluronan, cellulose, dextran, xanthan gum, konjac, and gelatin [40]. The use of synthetic polymers is rather limited due to the sensitive and fragile nature of the burn skin. However, synthetic polymers such as tri-block copolymers with ABA or BAB arrangements (e.g., polyethylene glycol (PEG)-poly(d,l-lactide-co-glycolide) (PLGA)-PEG, PLGA-PEG-PLGA) offer a unique advantage by aiding in the encapsulation and the delivery of poorly water soluble agents [41]. Hydrogels have been adopted in the various stages of burn wound management. For emergencies, hydrogels can help lower the temperature of the burn wound and deliver anesthetic agents [42]. Hydrogels can serve as primary wound dressings to accelerate wound healing and closure, additionally offering a skin generative template that minimizes scar formation and promotes the formation of skin appendages. Since the early eighties, hydrogels have been investigated in clinical settings. A number of hydrogel-based treatments in various forms (e.g., CVS Health Sterile Hydrogel Burn Pads) are commercially available in the treatment of superficial, cavity, and burn wounds [39]. In spite of various hydrogel products already on the market, hydrogels have been continuously researched and advanced from simple hydrogels composed of natural and/or synthetic polymers to complex, carefully engineered formulations such as skin-adaptable [43], stretchable [44], and sprayable hydrogels (Figure 2) [45]. In particular, research concerning probiotic hydrogels for burn wound healing has gained attention over the past decade due to the unique intrinsic properties that hydrogels can offer in burn wound treatments (Figure 2 and Figure 3).

**Table 2:** Advantages and disadvantages of drug delivery systems widely accepted for burn wound healing [46].

Form	Example	Description	Advantages	Disadvantages
Hydrogel	Skin-adaptable hydrogel dressing [45]	Hydrogels composed of a mixture of synthetic polymers	Skin-like, exude-absorbing, bioadhesive, cools the wounded area, offers pain-relief and moist environment, wearing comfort	Low storage stability (e.g., shrinking, swelling, syneresis), Need for preservatives
Ointment	Bacitracin ointment	Antimicrobial, petrolatum-based water-free	Inexpensive, can be applied to mucous membrane, occlusive	Does not penetrate eschar. May cause urticaria
Cream	Silver sulfadiazine (silvadene) cream	Interferes with bacterial DNA synthesis, water-based cream	Inexpensive, can be applied to mucous membrane	Need for frequent dressing changes, may need preservatives, limited eschar penetration
Gauze	Xeroform	Semi-occlusive, non-absorptive dressing	Non-adherent barrier, can serve as secondary dressing over absorptive dressing, clings to the body	Cannot be used for large exuding wounds, malodorous

Here, we summarized novel hydrogels loaded with probiotics such as injectable in situ hydrogels, emulgels (emulsion-gel), nanogels, acid-producing hydrogels, hydrogels crosslinked with polymeric micelles, bacteriomimetic hydrogel, and Chinese herbal medicine-based hydrogels and their wound healing properties.

Tao et al. prepared an injectable hydrogel that encapsulated live *Lactiplantibacillus plantarum* to combat pathogen wound infections by preventing biofilm formations [47]. The hydrogel was composed of hydrazide-functionalized gelatin and benzaldehyde-functionalized PEG. Each component was loaded in a separate cylinder and subsequently combined by pushing the merged plunger. The hydrazide group on the gelatin reacts with aldehydes on PEG to create hydrazones at neutral pH, initiating the gelation process. The authors stated that this “in situ” gelation offers a more precise control for administration and a capability of covering uneven surface of the wounds. Rheological testing revealed that gelation time was c.a. 60 s, indicating the immediate crosslinking process. Hydrogels loaded with *Lactiplantibacillus plantarum* (10^8^ CFU/mL) were effective in eradiating *Pseudomonas aeruginosa* and *Staphylococcus aureus*. The antifungal effect against *Candida albicans* was not as impressive as its antibacterial effect, still reducing 70% of *Candida albicans*. Hydrogels loaded with *Lactiplantibacillus plantarum* were tested with hemocompatibility as wound healing dressings are meant to be blood-contacting biomaterials. The test result shows that a hemolysis rate induced by hydrogels loaded with *Lactiplantibacillus plantarum* was <2% threshold. The application of hydrogels loaded with *Lactiplantibacillus plantarum* led to the prevention of biofilm formation by *Pseudomonas aeruginosa* and *Staphylococcus aureus*. Overall, this unique in situ hydrogel system was deemed safe and effective in managing wound infections.

Sharma et al. prepared gelatin emulgel by mixing Tween 80, glycerin, and cyclomethicone with gelatin followed by the addition of *Bacillus coagulans* spores (1.7 × 10^8^ CFU/g) dispersed in sunflower oil [48]. The authors observed a honeycomb-like network with void spaces and *Bacillus coagulans* embedded in the formulation which was viable even after being stored at various temperature and humidity conditions. The multiple applications of gelatin emulgel carrying *Bacillus coagulans* did not disrupt the epidermis, dermis, and hair follicles. The repeated application was performed topically for 28 days on female rats (2 × 10^8^ CFU/g). On the 28th day after application, the viable percentage of bacteria reduced to 0.05% and 0.5% in *Staphylococcus aureus* and *Pseudomonas aeruginosa*. The average wounded area on day 10 was c.a. 1 mm^2^ (control: 8 mm^2^), which shows superior wound reduction effect to that of marketed formulation (Soframycin^®^ containing Framycetin 1%, Sanofi, Paris, France) which showed c.a. 5 mm^2^ wound area. The authors stated that gelatin can aid wound healing itself by absorbing excess exudates from the wounds, providing homeostasis, and facilitating cell adhesion and proliferation during the wound healing process. Ultimately, gelatin emulgel encouraged the establishment of *Bacillus coagulans* and the subsequent germination to exert antibacterial effects.

Ashoori et al. developed chitosan nanogels carrying *Bacillus subtilis* sp. *natto* (B.S. natto, ATCC 15245), *Lactobacillus fermentum* (ATCC 9338), or *Lactobacillus reuteri* (ATCC 23272) strains for wound healing [49]. Chitosan nanogels were fabricated via ionic gelation of chitosan employing polyanion (i.e., sodium triphosphate). The average particle size of the chitosan nanogels was c.a. 25.6 nm, and the zeta potential was c.a. 13.07 mV. The wound healing rates were higher in the groups receiving the *Lactobacillus fermentum* supernatant-loaded nanogels and *Lactobacillus reuteri* supernatant-loaded nanogels. The wound healing process in these two groups was completed by day 10. The wound healing activity was shown to be insignificant when treated with empty chitosan nanogels. The authors referenced Han et al. [50] and Brandi et al. [51] and summarized that *Lactobacillus reuteri* enhances wound healing via the phosphoinositide 3 kinase (PI3K)/Akt/β-catenin/TGFβ1 pathway, and that *Lactobacillus fermentum* increases wound healing processes via exerting anti-inflammatory and antipathogenic influences. The authors claimed that chitosan offers improved healing effects by stimulating the liberation of inflammatory cytokines and proliferation of skin fibroblasts and keratinocytes.

Xu et al. utilized an extracellular polysaccharides (EPSs)-based hydrogel that releases lactic acid and acetic acid to resist the growth of pathogenic bacteria and preserve skin microbiota stability [3]. This hydrogel was composed of adhesion-enhanced EPSs (modified with dopamine) and hyaluronic acid methacrylate. The EPSs originated from *Bacillus velezensis*. These two components were crosslinked with hydrogen bonding followed by secondary covalent cross-linking at 405 nm. A probiotic strain of *Lacticaseibacillus paracasei* TYM202 (10^6^ CFU/mL) was loaded in the hydrogel to accelerate wound healing. This probiotic hydrogel was able to release lactic acid, acetic acid, propionic acid, and isovaleric acid and inhibit the growth of *Staphylococcus aureus* and *Escherichia coli* with a 2-fold increased healing rate on scratch assay as compared to empty hydrogels. In vivo results demonstrated that this probiotic hydrogel accelerated wound healing markedly faster compared to empty hydrogels. The authors also found that this probiotic hydrogel enhanced the level of M2 macrophages (anti-inflammatory macrophages) while significantly diminishing the level of M1 (pro-inflammatory macrophages). Anti-inflammatory factor IL-10 was also significantly higher and pro-inflammatory factor tumor necrosis factor-alpha (TNF-α) was much lower in the blood sample collected from the animals that received this probiotic hydrogel compared to other groups. The authors presumed that high concentrations of lactic acid released from the hydrogel inhibited glycolysis in immune cells, decreasing the rate of extracellular acidification and increasing the rate of oxygen consumption, which in turn, triggered an anti-inflammatory response to inhibit inflammation. The expression of the VEGF at the proliferative phase on day 9 in vivo was found to be much higher in the probiotic hydrogel-treated animals, indicating that this hydrogel promoted vascular regeneration. This probiotic hydrogel appeared to enhance collagen deposition and accelerate the regeneration of skin appendages. The authors performed 16S rRNA sequencing of the skin microbiota in both the control and probiotic hydrogel groups, comparing them with the original skin microbiota of rats. The probiotic-treated skin displayed similar skin microbiota compared to the original skin, whereas wounded skin that did not receive any treatment displayed lower *Firmicutes* and higher *Proteobacteria* at the phylum level. One of the notable findings was that the rat skin that received probiotic hydrogel treatment carried abundant *Lactobacilli* in the wound, at almost the same level as the original skin.

Mei et al. developed a probiotic hydrogel that can promote super bacteria-infected wound healing, which was composed of hyaluronate-adipic dihydrazone, aldehyde-terminated Pluronic F127/fucoidan micelle, and *Lactobacillus rhamnosus* (10^7^ CFU/mL) [4]. The crosslinking was made between the amine in hyaluronate-adipic dihydrazone and the aldehyde in aldehyde-terminated Pluronic F127/fucoidan micelle via the Schiff-base reaction. The authors found that the existence of probiotics accelerated the gelation of the hydrogels, which could be attributed to the free amino group in the probiotic cell wall interacting with aldehyde-terminated Pluronic F127/fucoidan micelle via the Schiff-base reaction. There was 100% antibacterial effect induced by this probiotic hydrogel against *Pseudomonas aeruginosa*, presumably due to the production of high levels of formic acid, acetic acid, and malic acid. The authors evaluated a wound healing effect on the rat skin wounded and infected with *Pseudomonas aeruginosa*. The probiotic hydrogel and Prontosan gel (commercially available, 0.1% polyaminopropyl biguanide, alkylamidopropyl betaine, purified water, hydroxyethylcellulose, and glycerol) exhibited wound healing effects on day 14 post-treatment. H&E and Masson trichrome staining of the skin showed much less inflammatory cell infiltration and more fibroblast migration when treated with the probiotic hydrogel. The rat skin treated with Prontosan gel failed to regenerate skin appendages and dermal tissues, whereas probiotic hydrogel-treated skin showed no difference from normal “unwounded” skin tissues.

Kuhn et al. developed a hydrogel containing bacteriomimetic microparticles [52]. First, membrane vesicles were harvested from *Lactobacillus plantarum* (p60 muropeptidase) and *Lacticaseibacillus casei* (p40 and p75 cell wall hydrolase) cultured in anaerobic and pH 6.5 conditions. Synthetic microparticles (aldehyde/sulfate latex beads) were then coated with these membrane vehicles and loaded in hydroxyethyl cellulose gels. This formulation induced elevated IL-10/TNF ratios, indicating the successful anti-inflammatory effect in vitro. This bacteriomimetic hydrogel assisted in quick reduction in wound width and length, indicating re-epithelialization, and appeared to dampen the inflammatory response during the wound healing process. It seems that the wound healing effect can be further improved by packing the membrane vesicles densely on the surface of the microparticle beads. The authors emphasized that bacteriomimetic hydrogels can potentially circumvent the issues related to the limited proliferation of microorganisms in immunocompromised patients.

Yang et al. developed a hydrogel containing *Lactobacillus plantarum* (10^10^ CFU/mL) utilizing oxidative polysaccharide-extracted *Bletilla striata* and chitosan [53]. *Bletilla striata*, also known as Hyacinth Orchid, is a traditional Chinese herb that is believed to be effective in wound healing, reducing bleeding and inflammation while promoting tissue regeneration. This hydrogel was biocompatible and swellable, reaching equilibrium at 4 h with a fluid absorption capacity of 3000–3500% in PBS. This demonstrates the capability of hydrogel to absorb the exudes from the wound. This hydrogel showed close to 100% antibacterial activities against *Staphylococcus aureus*, *Pseudomonas aeruginosa*, and *Escherichia coli*. The hydrogel also showed promising wound closure effect by demonstrating a c.a. 61% wound closure rate (c.a. 38% in control), an anti-inflammatory effect by decreasing the levels of TNF-alpha and IL-6, and a vascularization effect by increasing the level of VEGF. There was a more obvious, well-organized collagen deposition found in the wound area ex vivo when treated with this hydrogel.

## 4. Challenges and Future Perspective

The addition of known living beneficial cultures to products may cause concerns with microbial safety, especially in hydrogel formulations, due to the need for preservatives in formulations containing water. To avoid the need for preservatives, lyophilized, solid forms of probiotic hydrogels can be considered. Gruber et al. proved the effectiveness of STRATABIOSYS™ (Vantage Personal Care, New Jersey, USA), a preservative-free powder formulation that converts to a cream upon the addition of water, each containing 200 M CFU/gram of *Lactobacillus plantarum Lp90*, *Saccharomyces cerevisiae*, *Streptococcus thermophilus*, and *Lactococcus Lactis LLa61* [54]. This product was able to deliver probiotics to skin and upregulated 12–17 skin-relevant genes. Another option is to use heat-killed lysate; however, the living bacteria show superior benefits to the lysate in stimulating expression of key skin proteins including collagen [55,56]. It is also important to find the appropriate packaging that protects hydrogels from environmental moisture and minimizes a drying-out effect and syneresis (gel contraction followed by expulsion of water). Lastly, the development of rapid methods to measure skin microbial content/diversity and the effect of topically applied probiotic hydrogels must follow [57]. One of the simplest methods of measuring the effect on skin microbiome in a rapid manner is adenosine triphosphate (ATP) biofluorescence. Gruber et al. successfully examined the skin recovery (front arm) after 3% hydrogen peroxide treatment utilizing ATP biofluorescence. A pen-type surface ATP monitoring tool or a hand-held luminometer can measure the values of Relative Fluorescent Units (RFUs) indictive to the level of ATP attributed to skin microbiome. Comparing RFUs before and after treatments of topical probiotic hydrogels may offer insight into the impact of the treatments in skin microbiome and dysbiosis.

## 5. Conclusions

Probiotics not only promote gut health benefits but also show promising results in burn wound healing by offering antibacterial, anti-inflammatory, and re-epithelialization effects. Hydrogels, as a delivery system, can provide wide coverage of the wound surface and maintain moist healing environments which are the key factor in promoting rapid wound healing. Topical probiotic research on burn wound healing is still in its infancy although several studies have shown its effectiveness in vitro and in vivo. The dose-dependent effect on wound healing, more detailed research on the healing mechanisms, and the stability and permeability study of the loaded probiotics in and across the hydrogel matrices must be carried out to implement topical probiotic hydrogels as an alternative to conventional burn wound healing agents.

## Figures and Tables

**Table 1:** The summary of probiotic organisms known to manage skin disorders (modified from [8]).

Skin Disorder	Probiotic Organisms
Aging	*Nitrosomonas eutropha*, *Lactobacillus buchneri*
Acne	*Streptococcus thermophiles*, *Enterococcus faecalis*, *Streptococcus salivarlus*
Psoriasis	*Bifidobacteria infantis*, *Lactobacillus pentosus*
Atopic dermatitis	*Vitreoscilla filiformis*, *Streptococcus thermophilus*, *Lactobacillus johnsonii*, *Bifidobacterium species*
Wound healing	*Lactiplantibacillus plantarum*, *Kefir*, *Lactobacillus fermentum*, *Saccharomyces cerevisiae*
Rosacea	*Bifidobacterium breve BR03*, *Lactobacillus salivarius*

## References

[1] Carmona-Cruz S., Orozco-Covarrubias L., Sáez-de-Ocariz M. (2022). The Human Skin Microbiome in Selected Cutaneous Diseases. Front. Cell. Infect. Microbiol..

[2] Nakatsuji T., Chen T.H., Narala S., Chun K.A., Two A.M., Yun T., Shafiq F., Kotol P.F., Bouslimani A., Melnik A.V. (2017). Antimicrobials from Human Skin Commensal Bacteria Protect against *Staphylococcus aureus* and Are Deficient in Atopic Dermatitis. Sci. Transl. Med..

[3] Xu H., Li Y., Song J., Zhou L., Wu K., Lu X., Zhai X., Wan Z., Gao J. (2024). Highly Active Probiotic Hydrogels Matrixed on Bacterial EPS Accelerate Wound Healing via Maintaining Stable Skin Microbiota and Reducing Inflammation. Bioact. Mater..

[4] Mei L., Zhang D., Shao H., Hao Y., Zhang T., Zheng W., Ji Y., Ling P., Lu Y., Zhou Q. (2022). Injectable and Self-Healing Probiotics-Loaded Hydrogel for Promoting Superbacteria-Infected Wound Healing. ACS Appl. Mater. Interfaces.

[5] Byrd A.L., Belkaid Y., Segre J.A. (2018). The Human Skin Microbiome. Nat. Rev. Microbiol..

[6] Rozas M., Hart de Ruijter A., Fabrega M.J., Zorgani A., Guell M., Paetzold B., Brillet F. (2021). From Dysbiosis to Healthy Skin: Major Contributions of Cutibacterium Acnes to Skin Homeostasis. Microorganisms.

[7] Knackstedt R., Knackstedt T., Gatherwright J. (2020). The Role of Topical Probiotics on Wound Healing: A Review of Animal and Human Studies. Int. Wound J..

[8] Habeebuddin M., Karnati R.K., Shiroorkar P.N., Nagaraja S., Asdaq S.M.B., Anwer K., Fattepur S. (2022). Topical Probiotics: More Than a Skin Deep. Pharmaceutics.

[9] Ji J., Jin W., Liu S.-J., Jiao Z., Li X. (2023). Probiotics, Prebiotics, and Postbiotics in Health and Disease. MedComm.

[10] Mazziotta C., Tognon M., Martini F., Torreggiani E., Rotondo J.C. (2023). Probiotics Mechanism of Action on Immune Cells and Beneficial Effects on Human Health. Cells.

[11] Koirala S., Anal A.K. (2021). Probiotics-Based Foods and Beverages as Future Foods and Their Overall Safety and Regulatory Claims. Future Foods.

[12] Vinderola G., Sanders M.E., Salminen S. (2022). The Concept of Postbiotics. Foods.

[13] You S., Ma Y., Yan B., Pei W., Wu Q., Ding C., Huang C. (2022). The Promotion Mechanism of Prebiotics for Probiotics: A Review. Front. Nutr..

[14] Bouslimani A., da Silva R., Kosciolek T., Janssen S., Callewaert C., Amir A., Dorrestein K., Melnik A.V., Zaramela L.S., Kim J.-N. (2019). The Impact of Skin Care Products on Skin Chemistry and Microbiome Dynamics. BMC Biol..

[15] Davani-Davari D., Negahdaripour M., Karimzadeh I., Seifan M., Mohkam M., Masoumi S.J., Berenjian A., Ghasemi Y. (2019). Prebiotics: Definition, Types, Sources, Mechanisms, and Clinical Applications. Foods.

[16] Callewaert C., Knödlseder N., Karoglan A., Güell M., Paetzold B. (2021). Skin Microbiome Transplantation and Manipulation: Current State of the Art. Comput. Struct. Biotechnol. J..

[17] Żwierełło W., Piorun K., Skórka-Majewicz M., Maruszewska A., Antoniewski J., Gutowska I. (2023). Burns: Classification, Pathophysiology, and Treatment: A Review. Int. J. Mol. Sci..

[18] Church D., Elsayed S., Reid O., Winston B., Lindsay R. (2006). Burn Wound Infections. Clin. Microbiol. Rev..

[19] Maitz J., Merlino J., Rizzo S., McKew G., Maitz P. (2023). Burn Wound Infections Microbiome and Novel Approaches Using Therapeutic Microorganisms in Burn Wound Infection Control. Adv. Drug Deliv. Rev..

[20] Rowley-Conwy G. (2010). Infection Prevention and Treatment in Patients with Major Burn Injuries. Nurs. Stand..

[21] Macedo J.L.S.d., Santos J.B. (2005). Bacterial and Fungal Colonization of Burn Wounds. Mem. Inst. Oswaldo Cruz..

[22] Capoor M.R., Sarabahi S., Tiwari V.K., Narayanan R.P. (2010). Fungal Infections in Burns: Diagnosis and Management. Indian J. Plast. Surg..

[23] Radzikowska-Büchner E., Łopuszyńska I., Flieger W., Tobiasz M., Maciejewski R., Flieger J. (2023). An Overview of Recent Developments in the Management of Burn Injuries. Int. J. Mol. Sci..

[24] Lolou V., Panayiotidis M.I. (2019). Functional Role of Probiotics and Prebiotics on Skin Health and Disease. Fermentation.

[25] Bădăluță V.A., Curuțiu C., Dițu L.M., Holban A.M., Lazăr V. (2024). Probiotics in Wound Healing. Int. J. Mol. Sci..

[26] Satish L., Gallo P.H., Johnson S., Yates C.C., Kathju S. (2017). Local Probiotic Therapy with *Lactobacillus plantarum* Mitigates Scar Formation in Rabbits after Burn Injury and Infection. Surg. Infect..

[27] Peral M.C., Huaman Martinez M.A., Valdez J.C. (2009). Bacteriotherapy with *Lactobacillus plantarum* in Burns. Int. Wound J..

[28] Soleymanzadeh Moghaddam S., Momeni M., Mazar Atabaki S., Mousavi Shabestari T., Boustanshenas M., Afshar M., Roham M. (2022). Topical Treatment of Second-Degree Burn Wounds with *Lactobacillus plantarum* Supernatant: Phase I Trial. Iran. J. Pathol..

[29] Barzegari A.A., Hashemzaei M., Majdani R., Alihemmati A.-R. (2017). Effects of Topical Treatment of Second-Degree Burn Wounds with *Lactobacillus acidophilus* on the Wound Healing Process in Male Rats. Pharm. Biomed. Res..

[30] Jebur M. (2010). Therapeutic Efficacy of *Lactobacillus acidophilus* against Bacterial Isolates from Burn Wounds. N. Am. J. Med Sci..

[31] Khan H., Naeem N., Sughra S., Khan S., Soomro S.H., Abro A.S. (2023). Impact of Fermented Lactobacilli Acidophilus and Antibiotics Topically during the Phase of Re-Epithelization in Wound Repair of Rats. Prof. Med J..

[32] Oryan A., Alemzadeh E., Eskandari M.H. (2019). Kefir Accelerates Burn Wound Healing through Inducing Fibroblast Cell Migration In Vitro and Modulating the Expression of IL-1ß, TGF-SS1, and bFGF Genes In Vivo. Probiotics Antimicrob. Proteins.

[33] Rodrigues K.L., Caputo L.R.G., Carvalho J.C.T., Evangelista J., Schneedorf J.M. (2005). Antimicrobial and Healing Activity of Kefir and Kefiran Extract. Int. J. Antimicrob. Agents.

[34] Cetik Yildiz S., Demir C., Cengiz M., Ayhanci A. (2019). Protective Properties of Kefir on Burn Wounds of Mice That Were Infected with *S. aureus*, *P. auroginasa* and *E. coli*. Cell. Mol. Biol..

[35] Oryan A., Jalili M., Kamali A., Nikahval B. (2018). The Concurrent Use of Probiotic Microorganism and Collagen Hydrogel/Scaffold Enhances Burn Wound Healing: An In Vivo Evaluation. Burns.

[36] Zheng B.-D., Gan L., Tian L.-Y., Chen G.-H. (2023). Protein/Polysaccharide-Based Hydrogels Loaded Probiotic-Mediated Therapeutic Systems: A Review. Int. J. Biol. Macromol..

[37] Shu W., Wang Y., Zhang X., Li C., Le H., Chang F. (2021). Functional Hydrogel Dressings for Treatment of Burn Wounds. Front. Bioeng. Biotechnol..

[38] Yao Y., Zhang A., Yuan C., Chen X., Liu Y. (2021). Recent Trends on Burn Wound Care: Hydrogel Dressings and Scaffolds. Biomater. Sci..

[39] Madaghiele M., Sannino A., Ambrosio L., Demitri C. (2014). Polymeric Hydrogels for Burn Wound Care: Advanced Skin Wound Dressings and Regenerative Templates. Burn Trauma.

[40] Stoica A.E., Chircov C., Grumezescu A.M. (2020). Hydrogel Dressings for the Treatment of Burn Wounds: An Up-to-Date Overview. Materials.

[41] McKenzie M., Betts D., Suh A., Bui K., Kim L., Cho H. (2015). Hydrogel-Based Drug Delivery Systems for Poorly Water-Soluble Drugs. Molecules.

[42] He J.J., McCarthy C., Camci-Unal G. (2021). Development of Hydrogel-Based Sprayable Wound Dressings for Second- and Third-Degree Burns. Adv. NanoBiomed Res..

[43] Huangfu Y., Li S., Deng L., Zhang J., Huang P., Feng Z., Kong D., Wang W., Dong A. (2021). Skin-Adaptable, Long-Lasting Moisture, and Temperature-Tolerant Hydrogel Dressings for Accelerating Burn Wound Healing without Secondary Damage. ACS Appl. Mater. Interfaces.

[44] George B., Bhatia N., Kumar A., Gnanamani A., Thilagam R., Shanuja S.K., Vadakkadath Meethal K., Shiji T.M., Suchithra T.V. (2022). Bioinspired Gelatin Based Sticky Hydrogel for Diverse Surfaces in Burn Wound Care. Sci. Rep..

[45] Choi J.Y., Joo Y.-J., Kang R.J., Jeon H.K., Hong G.S. (2024). Effect of Spray-Type Alginate Hydrogel Dressing on Burn Wounds. Gels.

[46] Lanham J.S., Nelson N.K., Hendren B., Gordon F., Jordan T.S. (2020). Outpatient Burn Care: Prevention and Treatment. Am. Fam. Physician.

[47] Tao S., Zhang S., Wei K., Maniura-Weber K., Li Z., Ren Q. (2024). An Injectable Living Hydrogel with Embedded Probiotics as a Novel Strategy for Combating Multifaceted Pathogen Wound Infections. Adv Healthc. Mater..

[48] Sharma G., Sharma M., Sood R., Neelamraju J., Lakshmi S.G., Madempudi R.S., Rishi P., Kaur I.P. (2021). Self-Preserving Gelatin Emulgel Containing Whole Cell Probiotic for Topical Use: Preclinical Safety, Efficacy, and Germination Studies. Expert Opin. Drug Deliv..

[49] Ashoori Y., Mohkam M., Heidari R., Abootalebi S.N., Mousavi S.M., Hashemi S.A., Golkar N., Gholami A. (2020). Development and In Vivo Characterization of Probiotic Lysate-Treated Chitosan Nanogel as a Novel Biocompatible Formulation for Wound Healing. BioMed Res. Int..

[50] Han N., Jia L., Su Y., Du J., Guo L., Luo Z., Liu Y. (2019). *Lactobacillus reuteri* Extracts Promoted Wound Healing via PI3K/AKT/β-Catenin/TGFβ1 Pathway. Stem Cell Res. Ther..

[51] Brandi J., Cheri S., Manfredi M., Di Carlo C., Vita Vanella V., Federici F., Bombiero E., Bazaj A., Rizzi E., Manna L. (2020). Exploring the Wound Healing, Anti-Inflammatory, Anti-Pathogenic and Proteomic Effects of Lactic Acid Bacteria on Keratinocytes. Sci. Rep..

[52] Kuhn T., Aljohmani A., Frank N., Zielke L., Mehanny M., Laschke M.W., Koch M., Hoppstädter J., Kiemer A.K., Yildiz D. (2024). A Cell-Free, Biomimetic Hydrogel Based on Probiotic Membrane Vesicles Ameliorates Wound Healing. J. Control. Release.

[53] Yang L., Han Z., Chen C., Li Z., Yu S., Qu Y., Zeng R. (2020). Novel Probiotic-Bound Oxidized *Bletilla striata* Polysaccharide-Chitosan Composite Hydrogel. Mater. Sci. Eng. C.

[54] Gruber J.V., Holtz R., Roach M. (2024). Examining the Genomic Influence of Topically Applied Probiotics In Vitro. Int. J. Cosmet. Sci..

[55] Khmaladze I., Butler É., Fabre S., Gillbro J.M. (2019). Lactobacillus reuteri DSM 17938—A Comparative Study on the Effect of Probiotics and Lysates on Human Skin. Exp. Dermatol..

[56] Garlet A., Leoty-Okombi S., Gault M., Aversa L., Pelletier N., Bonnaud-Rosaye C., Chan W., Andre V. (2022). 524 Better Performance of Live Probiotic over Inactivated Biomass on Skin Density. J. Investig. Dermatol..

[57] Gruber J.V., Riemer J. (2022). Examining Skin Recovery after a 3% Aqueous Hydrogen Peroxide (H_2_O_2_) Treatment Using ATP Biofluorescence. Clin. Cosmet. Investig. Dermatol..

